# Estradiol ameliorates arthritis and protects against systemic bone loss in *Staphylococcus aureus *infection in mice

**DOI:** 10.1186/ar3799

**Published:** 2012-04-16

**Authors:** Inger Gjertsson, Marie K Lagerquist, Erik Kristiansson, Hans Carlsten, Catharina Lindholm

**Affiliations:** 1 Department of Rheumatology and Inflammation Research, Institute of Medicine, Guldhedsgatan 10A, Gothenburg, S-413 46, Sweden; 2 CBAR, Centre for Bone and Arthritis Research, Sahlgrenska Academy at University of Gothenburg, Guldhedsgatan 10A, Gothenburg, S-413 46, Sweden; 3 Department of Mathematical Statistics, Chalmers University of Technology, Chalmers tvärgata 3, Gothenburg, S-412 96, Sweden

## Abstract

**Introduction:**

*Staphylococcus aureus *is a common cause of bacterial arthritis, which is associated with progressive bone loss in affected joints. We recently showed that *S. aureu*s infection also induces a significant systemic bone loss in mice. This study was performed to assess the effect of estradiol treatment on the clinical course and outcome of *S. aureus *arthritis and on infection-induced bone loss in experimental *S. aureus *infection.

**Methods:**

Mice were ovariectomized, treated with estradiol or placebo, and *S. aureus *infection was established by intravenous inoculation of bacteria.

**Results:**

Estradiol treatment was found to decrease significantly the frequency and clinical severity of *S. aureus *arthritis, a finding that was accompanied with significantly higher serum levels of interleukin-10 in estradiol-treated mice. Estradiol was also highly protective against *S. aureus*-induced systemic trabecular, and cortical bone loss. Lack of endogenous estrogens and *S. aureus *infection had additive effects on trabecular bone loss. The *S. aureus-*infected, ovariectomized mice lost as much as 76% of their trabecular bone mass.

**Conclusions:**

Treatment with estradiol ameliorates *S. aureus *arthritis and is protective against infection-induced systemic bone loss in experimental *S. aureus *infection.

## Introduction

Bacterial arthritis is a disease with high mortality and morbidity characterized by severe, progressive joint destruction that occurs despite antibiotic treatment. *Staphylococcus aureus *is one of the most common causative pathogens in bacterial arthritis [1], and patients with underlying joint diseases (for example, rheumatoid arthritis, or taking immunosuppressive therapy) are at higher risk for this infection [2]. In a mouse model of hematogenously spread *S. aureus *infection, we showed that the infection, in addition to joint destruction, also induces pronounced systemic bone loss within a few days after bacterial inoculation [3]. Even though it is well known that septic arthritis causes a dramatic bone loss locally in the affected joints in humans, it remains to be investigated whether systemic bone loss is seen in patients with severe bacterial infections, including septic arthritis. The *S. aureus*-induced bone loss is likely to be mediated by osteoclasts, the only known cell type with bone-resorptive capacity, which can be activated by different proinflammatory cytokines (for example, tumor necrosis factor (TNF)-α and interleukin (IL)-6) [4,5]. Both these proinflammatory cytokines are increased at the systemic level of *S. aureus-*infected mice [6,7], and TNF-α shows an increased gene expression in the synovium of infected joints [8]. Furthermore, we showed that TNF-α [9] and interferon (IFN)-γ [6] strongly contribute to *S. aureus*-induced joint destruction. The antiinflammatory cytokine IL-10 plays an important role for control of excessive immunity and damage to the host during infections [10], and we showed that IL-10 is protective against *S. aureus *arthritis, at least partly by inhibition of TNF-α [11]. In addition, IL-10 has been demonstrated to inhibit osteoclast differentiation *in vitro *[12] and to suppress infection-induced bone resorption in a murine model of periodontitis [13].

Our previous studies showed that osteoclast-inhibiting treatment with bisphosphonates or receptor activator of nuclear factor-κB ligand (RANKL)-targeted therapy reduce the systemic bone loss in *S. aureus *arthritis in mice [14,15]. In contrast, the joint destruction was not affected by these osteoclast-inhibiting therapies.

The female sex hormone estradiol is bone protective and is likely to prevent osteoporosis both by direct effects on bone-resorbing osteoclasts and bone-forming osteoblasts and by immunomodulatory effects changing the cytokine milieu in the bone compartment. Estradiol treatment can ameliorate autoimmune arthritis and joint destruction both in humans and in mice [16-18], and we previously showed that treatment with estradiol decreases both the systemic and local bone loss in autoimmune arthritis in mice [19,20].

The aim of this study was therefore to evaluate the effect of estradiol on the course and outcome of *S. aureus *infection, as well as on local and systemic bone loss in infected mice. Our results clearly show that treatment with estradiol ameliorates *S. aureus *arthritis and protects against *S. aureus*-induced systemic bone loss.

## Materials and methods

### Mice

Female C57 BL/6 mice were maintained in the animal facility at the Department of Rheumatology and Inflammation Research at Göteborg University under standard light and temperature conditions. Mice were fed laboratory chow and tap water *ad libitum*. Permission from the local animal research ethics committee, in accordance with national animal welfare legislation, was obtained for all the mice experiments.

### Ovariectomy and estrogen treatment

Ovariectomy or sham operations were performed at 10 weeks of age under isoflurane anesthesia, and carprofen (OrionPharma AB, Sollentuna, Sweden) was used as postoperative analgesia. Estradiol treatment was started at ovariectomy. Slow-release pellets (Innovative Research of America, Sarasota, FL, USA) containing 0.05 mg 17β-estradiol per pellet were implanted subcutaneously, giving a release of 0.8 μg 17β-estradiol per 24 hours. As a treatment, control mice were given placebo-containing subcutaneous pellets (Innovative Research of America).

### Mouse model of systemic *S. aureus *infection and arthritis

The TSST-1 producing *S. aureus *strain LS-1, originally isolated from a spontaneously arthritic NZB/W mouse [21], was used for induction of septic arthritis. Bacteria were grown overnight, harvested, and resuspended in phosphate-buffered saline (PBS) containing 5% bovine serum albumin and 10% dimethylsulfoxide and kept in aliquots at -20°C until use. The number of colony-forming units (CFUs) of the bacterial suspension was determined by performing repeated viable counts. Before use, bacteria were thawed, washed in PBS, and diluted to appropriate concentration by calculation with the previously determined CFUs. Viable counts were performed to determine the actual number of viable bacteria given in each experiment. One week after ovariectomy, mice were inoculated intravenously with 7 to 8 × 10^7 ^CFU/mouse of *S. aureus *LS-1 in 200 μl of PBS in one of the tail veins.

All mice were followed up individually and checked daily. Mice were graded blindly for arthritis severity and frequency. Finger/toe and ankle/wrist joints were inspected, and arthritis was defined as visible erythema and/or swelling. To evaluate the intensity of arthritis, a clinical scoring (arthritic index) was carried out by using a system in which macroscopic inspection yielded a score of 0 to 3 points for each limb (0, neither swelling nor erythema; 1, mild swelling and/or erythema; 2, moderate swelling and erythema; and 3, marked swelling and erythema). The total score was calculated by adding all the scores for each animal tested.

The overall condition of each mouse was examined daily by assessing signs of systemic inflammation (that is, weight decrease, reduced alertness, and ruffled coat). In cases of severe systemic infection, when a mouse was judged too ill to survive another 24 hours, it was culled and defined as dead due to sepsis.

Mice were killed, and blood, kidneys, and limbs were obtained on days 3, 7, and 14 after bacterial inoculation.

### Histology of inflamed joints

Histopathologic examination of the joints was performed after routine fixation, decalcification, and paraffin embedding. Tissue sections from fore- and hindpaws were cut and stained with hematoxylin-eosin. All the slides were coded and evaluated by two blinded observers. The specimens were evaluated with regard to synovial hypertrophy/infiltration of leukocytes and cartilage/subchondral bone erosion. The degree of synovitis and erosion yielded a score from 0 to 3 in every joint, concerning finger/toes, wrists/ankles, elbows and knees. Occasionally one paw was missing in the histologic sections, or embedded in a way that made it impossible to evaluate the degree of synovitis and bone/cartilage erosion, and therefore, the total score/mouse is divided by the number of joints evaluated.

### Bacteriologic examination

The bacterial content in kidneys was determined at sacrifice. The kidneys were aseptically removed, mechanically homogenized, and diluted in 10 ml of PBS. Viable counts were performed by culturing the tissue suspension on horse-blood agar plates overnight to determine the number of *S. aureus *CFUs.

### Assessment of bone mineral density

One femur from each mouse was subjected to peripheral quantitative computed tomography (pQCT) scan with Stratec pQCT XCT Research M, software version 5.4 B (Norland, Fort Atkinson, WI, USA) at a resolution of 70 μm, as previously described [22]. Trabecular bone mineral density (BMD) was determined with a metaphyseal scan, which was performed at a distance from the growth plate corresponding to 3% of the length of the femur. The inner 45% of the area was defined as the trabecular bone compartment. Cortical BMD was determined with a mid-diaphyseal scan located 36% of the length of the femur from the growth plate.

### Cytokine analysis

Serum levels of IL-6, IL-10, IL-17A, interferon (IFN)γ, and TNF-α were determined by using cytometric bead assay (CBA) mouse Th1/Th2/Th17 kit (BD Biosciences, Erebodegem, Belgium) according to the manufacturer's instructions. Samples were run on a FACSCanto II (BD Biosciences). Detection limits were 1.4, 16.8, 0.8, 0.5, and 0.9 pg/ml for IL-6, IL-10, IL-17A, IFN-γ, and TNF-α, respectively.

### Analysis of serologic markers of bone and cartilage remodeling

Serum levels of fragments of type I collagen were analyzed as markers of bone resorption by using a RatLaps ELISA kit (Nordic Bioscience Diagnostics A/S, Herlev, Denmark) according to the manufacturer's instructions. As a marker of cartilage degradation, serum levels of cartilage oligomeric matrix protein (COMP) were determined by using an animal COMP ELISA assay (AnaMar Medical AB, Gothenburg, Sweden).

### Statistical evaluations

Statistical analyses were performed by using GraphPad Prism (La Jolla, CA, USA) and R 2.10.1 [23]. Statistical association between frequency of arthritis and treatment was tested by using the Fisher Exact test. The change in severity of clinical arthritis between treatments was tested by using robust linear regression from the MASS R-package. Differences in cytokine levels between independent groups were tested by using Kruskal-Wallis tests with the Dunn correction for multiple comparisons, whereas two-way analyses of variance (ANOVAs) with Bonferroni posttests were used to assess the combined effect of hormones and infection on BMD. Survival analysis was performed by using the Kaplan-Meier model, and differences in survival were tested by using the log-rank test. A *P *value of ≤ 0.05 was considered statistically significant.

## Results

### Estradiol ameliorates development and severity of *S. aureus-*induced arthritis

The effect of estradiol treatment on the development and severity of *S. aureus-*induced arthritis was studied by inoculating ovariectomized mice treated with estradiol or placebo with *S. aureus *intravenously.

**Figure 1:**
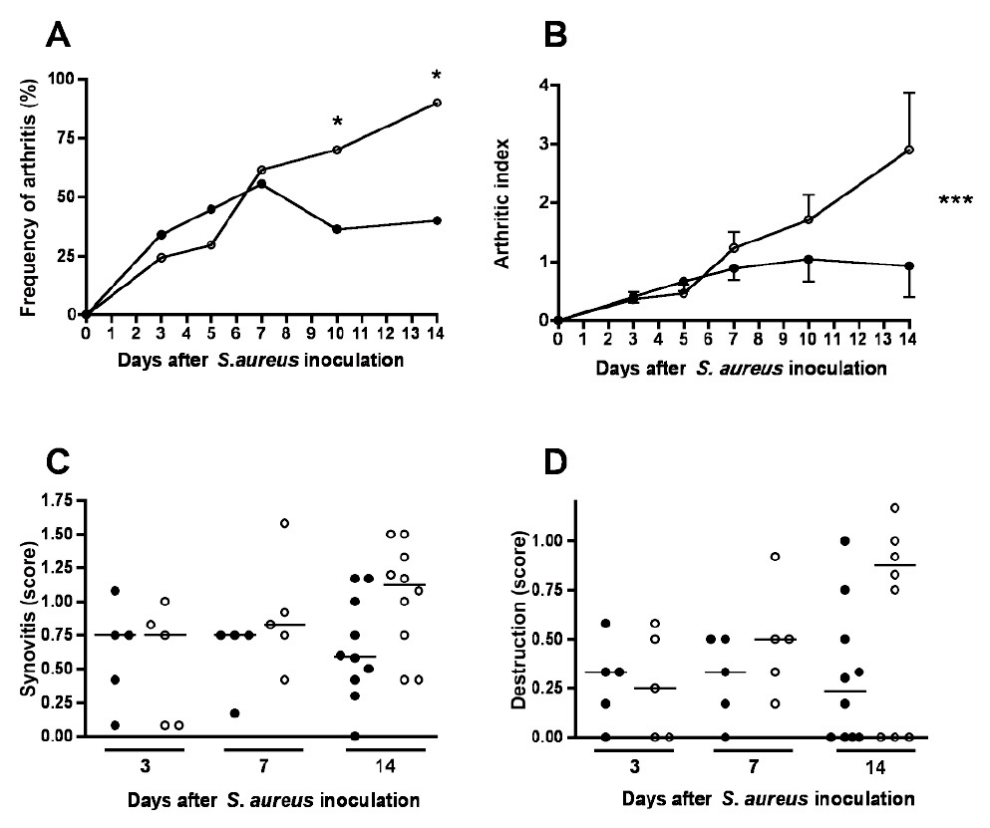
**Effect of estradiol treatment on clinical course of *Staphylococcus aureus *infection**. Frequency of arthritis **(A)**, clinical severity of arthritis **(B)**, histologic evaluation of synovitis **(C) **and erosion **(D) **of bone and cartilage in joints, as evaluated microscopically. Solid circles, estradiol-treated mice; open circles: placebo-treated mice. Values in B are given as mean ± SEM. Horizontal bars in C and D show median values, and each symbol represents the histologic score for one mouse. Data in A and B are pooled from two separate experiments with 12 to 14 mice per treatment group. *P < 0.05, as analyzed with the Fisher Exact test; ***P < 0.001, as analyzed with regression analysis.

Development of *S. aureus *arthritis was significantly inhibited by estradiol treatment; at day 10 after bacterial inoculation, only eight (36%) of 22 estradiol-treated mice had developed arthritis, as compared with 14 (70%) of 20 in the placebo group, and at day 14, the frequency of arthritis was only 40% (four of 10) in the estradiol-treated group, as compared with 90% (nine of 10) in the placebo-treated mice (Figure 1A). In addition, estradiol-treated mice had significantly less severe clinical arthritis as compared with placebo-treated mice (Figure 1B). Histopathologic evaluation of the joints did not show any statistically significant reduction of synovitis or joint destruction in estradiol-treated mice compared with the placebo-treated group (Figure 1C, D).

To evaluate whether estradiol affected the general outcome of *S. aureus *infection, the mortality, weight change (as a sign of general health), and bacterial persistence were studied.

No significant differences in survival rates were found between estradiol-treated (20 of 26 mice, 74%) and placebo-treated (19 of 25 mice, 74%) groups. The weight loss was not significantly different at any time in estradiol- as compared with placebo-treated mice (not shown). Bacterial persistence in host tissue (kidneys) was not affected by estradiol treatment; at day 14 after bacterial inoculation, the median CFUs/two kidneys was 4.1 × 10^6 ^(range, 0 to 4.9 × 10^7^) and 2.7 × 10^6 ^(range, 0 to 2.2 × 10^7^) in estradiol- and placebo-treated mice, respectively. Similarly, bacterial numbers in kidneys obtained on days 3 and 7 did not differ between estradiol- or placebo-treated mice (not shown).

### Estradiol treatment protects against systemic bone loss in *S. aureus *infection

**Figure 2:**
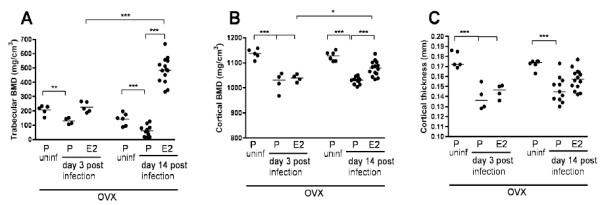
**Effect of estradiol treatment on *Staphylococcus aureus*-induced systemic bone loss as analyzed with peripheral quantitative computed tomography (pQCT)**. Trabecular bone mineral density (BMD) **(A)**, cortical BMD **(B)**, and cortical thickness **(C) **in ovariectomized estradiol- and placebo-treated *S. aureus-*infected mice and in ovariectomized, placebo-treated, uninfected controls. Horizontal bars show median values, and each symbol represents one mouse. *P < 0.05; **P < 0.01; and ***P < 0.001; as analyzed with two-way ANOVAs with Bonferroni posttests.

**Figure 3:**
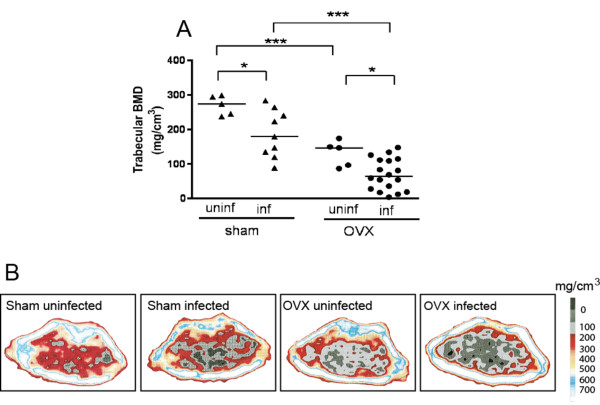
**Relative contribution of ovariectomy and *Staphylococcus aureus *infection on bone loss as analyzed with peripheral quantitative computed tomography (pQCT)**. Trabecular bone mineral density (BMD) **(A) **and representative pQCT images **(B) **at day 14 after intravenous bacterial inoculation in sham-operated and ovariectomized *S. aureus-*infected mice. Samples from uninfected mice were taken at the same time. Horizontal bars show median values. Solid triangles, sham-operated mice; solid circles, ovariectomized mice. Each symbol represents one mouse. *P < 0.05; **P < 0.01; ***P < 0.001; as analyzed by two-way ANOVA with Bonferroni posttests.

To study the effect of estradiol on *S. aureus*-induced systemic bone loss, trabecular and cortical bone BMD and cortical thickness were evaluated with pQCT. *S. aureus-*infected, ovariectomized, placebo-treated mice developed significant loss of trabecular bone (Figures 2A and 3A). Estradiol treatment totally prevented the *S. aureus*-induced trabecular bone loss, and at day 14 after bacterial inoculation, the trabecular BMD had increased significantly as compared with day 3 (Figure 2A). The median trabecular BMD of 487 mg/cm^3 ^at day 14 after *S. aureus *inoculation of estradiol-treated mice was significantly lower than the trabecular BMD of 588 mg/cm^3 ^in estradiol-treated uninfected mice at this time (*P *< 0.05, not shown). Estradiol treatment also had a clear bone-protective effect on cortical BMD, with significantly higher cortical BMD in the estradiol- as compared with the placebo-treated group at day 14 (Figure 2B). Cortical BMD was significantly increased at day 14, as compared with day 3, in estradiol-treated mice (Figure 2B). The *S. aureus *infection induced a significant reduction of cortical thickness in placebo- and estradiol-treated mice at day 3 and in placebo-treated mice at day 14 (Figure 2C). Estradiol had no significant effect on thickness of cortical bone.

Analyses of serum levels of bone and cartilage turnover markers showed that the levels of the bone-resorption marker RatLaps (type I collagen cross-links) increased significantly during the infection in placebo-treated but not in estradiol-treated mice (not shown). The cartilage-turnover marker COMP was not significantly increased after *S. aureus *infection, median level in uninfected, ovariectomized mice being 2.1 U/L (range, 1.8 to 2.2 U/L), as compared with 2.5 U/L (range, 2.2 to 2.9 U/L) in infected, ovariectomized mice or influenced by estradiol treatment.

### Serum levels of IL-10 are increased in estradiol-treated *S. aureus-*infected mice

To evaluate whether the protective effect of estradiol on clinical signs of *S. aureus*-induced arthritis and bone loss could be mediated by modulation of the systemic cytokine responses, we analyzed the serum protein levels of the proinflammatory cytokines TNF-α, IL-6, IFN-γ, and IL-17A and the antiinflammatory IL-10. Estradiol treatment of infected mice induced significantly increased serum protein levels of IL-10 at day 14 compared with placebo-treated mice (Table 1). The serum protein levels of TNF-α were significantly increased at day 3 after bacterial inoculation in placebo-treated mice compared with uninfected mice (Table 1). The serum levels of IFN-γ and IL-17A were low and were not affected by the infection or by the estradiol treatment.

**Table 1:** Serum cytokine levels in *Staphylococcus aureus*-infected mice treated with estradiol or placebo and in uninfected, ovariectomized control mice

		*S. aureus *infected	
		
Cytokine^a^	Uninfected (*n *= 12)	Placebo (*n *= 9)	Estradiol (*n *= 8)
IL-10	16.8 (16.8-16.8)	8.4 (8.4-16.8)	18.2 (12.6-172.4)^bc^
TNF-α	5.7 (3.9-8.7)	18.4 (15.0-23.6)^d^	20.4 (15.0-32.1)
IL-6	3.3 (1.7-92.3)	49.4 (24.6-454.8)	49.3 (13.3-617.0)
IFN-γ	1.2 (0.5-1.5)	1.6 (1.5-2.4)	2.0 (1.1-3.8)
IL-17A	1.2 (0.8-1.7)	1.8 (1.6-3.0)	2.6 (1.3-8.0)

^a^Values in pictograms per milliliter are given as medians (interquartile ranges). All mice were ovariectomized. Uninfected mice were treated with placebo. Infected mice were killed at day 14 after bacterial inoculation. ^b^*P *< 0.05, as compared with infected, placebo-treated mice. ^c^*P *< 0.01; ^d^*P *< 0.001, as compared with uninfected controls. Statistical analyses were done with the Kruskal-Wallis test followed by Dunn multiple-comparison test.

### *S. aureus *infection and lack of endogenous estrogens have an additive effect on systemic trabecular bone resorption

Because both lack of endogenous estrogens and *S. aureus *infection can induce systemic bone loss, we evaluated the relative contribution of lack of endogenous estrogens, as induced by ovariectomy, and *S. aureus *infection on BMD, as measured with pQCT. In the presence of endogenous estrogens (sham-operated mice), *S. aureus *infection induced an average decrease of trabecular BMD of 34% (that is, the median trabecular BMD was 180 mg/cm^3 ^at day 14 after bacterial inoculation as compared with 274 mg/cm^3 ^in uninfected mice) (Figure 3A, B). In ovariectomized mice (lacking endogenous estrogens), the average decrease of trabecular BMD was 56% (that is, 65 mg/cm^3 ^in infected mice at day 14 as compared with 147 mg/cm^3^in uninfected mice (Figure 3A, B). Ovariectomy alone induced an average decrease of trabecular BMD of 46% at day 21 after ovariectomy (Figure 3A, B). Lack of endogenous estrogens and *S. aureus *infection had a clear additive effect on trabecular bone loss. Thus, ovariectomized, *S. aureus*-infected mice showed a dramatic decrease of trabecular BMD, on average, -76%, compared with sham-operated, uninfected mice (Figure 3A, B). Cortical BMD was not affected by *S. aureus *infection of sham-operated mice, but was significantly decreased in *S. aureus-*infected, ovariectomized mice (not shown).

## Discussion

*S. aureus *arthritis is a severe disease associated with significant bone destruction that occurs despite antibiotic treatment. Thus new treatment strategies are needed to prevent the infection-induced bone loss. Estradiol has been shown to ameliorate nonseptic arthritis and to protect against inflammation-induced bone loss in both humans [16,24] and mice [17,19]. However, it has not been investigated whether estradiol treatment can be protective against septic arthritis or infection-induced bone loss. Our study is the first to show that estradiol treatment ameliorates the development of arthritis and protects against systemic bone loss in *S. aureus *infection in mice.

We found that estradiol treatment strongly reduced both the frequency and severity of *S. aureus*-induced arthritis at a later stage of *S. aureus *infection, importantly without affecting the bacterial clearance. These findings suggests an immunomodulatory role of estradiol in septic arthritis similar to the immune-dampening effects of estradiol previously demonstrated by us and others in nonseptic, autoimmune arthritis in humans and mice [16,17,19]. The antiarthritic effect of estradiol in the present study was of a comparable magnitude with treatment with antibiotics in our mouse model of *S. aureus *arthritis [14,25]. Our data demonstrate that estradiol-mediated suppression of the immune response during *S. aureus *infection is beneficial for the host. Even though we did not use antibiotic treatment in the present study, one might expect an even better outcome if estradiol had been combined with antibiotics.

In the present study, we demonstrated that estradiol totally prevented the pronounced systemic loss of trabecular bone noted in our previous studies of *S. aureus *infection in mice [14,15]. The trabecular BMD was preserved in estradiol-treated, infected mice at day 3 after bacterial inoculation and increased throughout the experiment, in contrast to the placebo-treated mice, which had a significant decrease of trabecular BMD 3 days after bacterial inoculation, with continuous loss of trabecular BMD during the study period. The higher trabecular BMD seen in estradiol-treated mice at day 14 as compared with day 3 was likely to reflect that the peak of the estradiol effect in this bone compartment was not reached until the later time. Estradiol was also protective against decrease of cortical BMD, but only at a later stage of the infection, with significantly higher cortical BMD in the estradiol-treated mice at day 14 after *S. aureus *inoculation. These temporal differences of the protective effects of estradiol in the trabecular and cortical bone compartments is likely explained by the higher bone-remodeling rate in trabecular bone, rendering this compartment more responsive to factors affecting bone metabolism.

Collectively, these bone-protective effects of estradiol in *S. aureus *infection are in good accord with our previous studies showing a bone-protective effect of estrogen in nonseptic, autoimmune arthritis in mice [19,20].

Our data show that the serum levels of IL-10 are increased in estradiol-treated mice. It is known that estradiol induces IL-10 production [26-28] and that IL-10 can inhibit osteoclast activity and differentiation in mice and humans [12,29,30]. Thus, it is likely that the increased serum levels of IL-10 in estradiol-treated mice contribute to the protective effect of estradiol on clinical development of arthritis as well as on systemic bone loss. Interestingly, estradiol was recently shown to increase regulatory T cell-mediated suppression of osteoclast differentiation and bone resorption *in vitro *via induction of IL-10 and TGF-β production [31]. Our finding of increased IL-10 levels at later stages of *S. aureus *infection suggests that the cellular source of estradiol-induced IL-10 might be regulatory T cells, but this remains to be investigated. Finally, we previously showed that IL-10 ameliorates *S. aureus *arthritis in mice [11], and others demonstrated that estradiol ameliorates adjuvant-arthritis in rodents in conjunction with an increased IL-10 response [32]. Another possible mechanism behind the increased IL-10 levels is induction of IL-10 production by proinflammatory cytokines (for example, TNF-α and IL-6). However, we did not find any correlation between the levels of IL-10 and TNF-α on an individual basis indicating independence between these cytokine responses, but further studies are needed.

Even though we found a significant effect of estradiol treatment on clinical signs of arthritis, supporting an antiinflammatory effect of estradiol treatment, in our model, the effects on BMD might of course also be due to the anabolic effect of estradiol on bone.

The role of estrogens in host defense against infections is not fully understood, as reviewed by Marriott *et al. *[33]. Estradiol treatment in our study did not influence the host ability to clear bacteria from infected tissues and general signs of bacterial burden (for example, weight loss or mortality were very similar in estradiol and placebo-treated groups).

In contrast to the clear antiresorptive effect of estradiol on systemic bone, we did not find any significant reduction of joint destruction in estradiol-treated mice, even though a tendency was present for a protective role of estradiol. This is also in contrast to our previous reports on a strong protective effect of estradiol on joint destruction in nonseptic, collagen-induced arthritis in mice [19]. Several reasons are possible for this discrepancy. First, the joint destruction might be caused by different mechanisms at the cellular and/or molecular level in septic and nonseptic arthritis. Second, the local levels of inflammatory cytokines might be higher in the joints of *S. aureus*-infected mice than in nonseptic arthritic joints exceeding the immunosuppressive capacity of estradiol. Third, the capacity of *S. aureus *exists to activate osteoclasts directly or indirectly via increased RANKL expression on osteoblasts [34,35]. However, we could not demonstrate any reduction of joint destruction in a previous study using RANKL-targeted therapy in *S. aureus *arthritis in mice [14,15].

Interestingly, our results show that a postmenopausal state, induced by ovariectomy, and *S. aureus *infection have strong additive effects on loss of trabecular bone (that is, ovariectomized and infected mice showed an average decrease of 76% of their trabecular BMD compared with sham-operated uninfected mice).

It is not known whether severe *S. aureus *infections, or other bacterial systemic infections, induce systemic bone loss, and thereby increase risk for osteoporosis and related fractures, in patients in general or in postmenopausal women in particular. However, it has been clearly demonstrated that chronic inflammation, such as in rheumatoid arthritis, and a postmenopausal state have an additive effect on bone loss and osteoporosis. The frequency of osteoporosis is approximately 50% in postmenopausal women with RA [36,37]. Our finding of a dramatic infection-induced systemic bone loss in experimental menopause warrants further studies of bone loss in postmenopausal women with severe bacterial infections.

## Conclusions

In conclusion, our study of arthritis and bone loss in *S. aureus *infection in mice provides evidence for an antiarthritic and strong bone-protective role of estradiol in infectious arthritis. Our findings may have future implications for the treatment of bacterial arthritis with estrogens or selective estrogen-receptor modulators, SERMs in combination with antibiotics.

## Abbreviations

BMD: bone mineral density; CFU: colony-forming unit; COMP: cartilage oligomeric matrix protein; ELISA: enzyme-linked immunosorbent assay; IL: interleukin; IFN: interferon; OVX: ovariectomy; PBS: phosphate-buffered saline; pQCT: peripheral quantitative computed tomography; RANKL: receptor activator of nuclear factor-κB ligand; TNF: tumor necrosis factor; TSST: toxic shock syndrome toxin.

## Competing interests

The authors declare that they have no competing interests.

## Authors' contributions

IG, MKL, and CL participated in the study design, writing of the manuscript, and data analysis, and performed the experiments. HC participated in the study design and writing of the manuscript. EK participated in data analysis and writing of the manuscript. All authors read and approved the manuscript.
